# Identification of Viral Variants from Functional Genomics Data

**DOI:** 10.12688/f1000research.168786.2

**Published:** 2025-12-31

**Authors:** Florian Röckl, Caroline C. Friedel

**Affiliations:** 1 Institute for Informatics, Ludwig-Maximilians-Universitaet Muenchen (LMU), Munich, Bavaria, Germany

**Keywords:** variant calling pipeline, functional genomics data, virus infections, null mutant virus

## Abstract

**Background:**

Virus mutants are commonly used for studying the role of individual viral proteins in infections and are increasingly investigated with functional genomics experiments of infected cells that use sequencing-based assays such as RNA-seq or ATAC-seq. However, existing mutant virus strains are often poorly documented, in particular if they have been created decades ago. Identifying viral variants directly in the functional genomics experiments avoids additional genome sequencing and allows confirming the presence of specific mutations directly in the experiment of interest.

**Methods:**

We present a pipeline to directly identify mutations in viral genomes from sequencing-based functional genomics data. The pipeline combines existing SNP callers with novel methods for identifying deletions, insertions, and corresponding inserted sequences. These novel methods address the problem that existing structural variant callers performed poorly on functional genomics data with large variations in read coverage.

**Results:**

We evaluated the pipeline on RNA-seq data for infection with knockout mutants for important proteins of Herpes simplex virus 1 (HSV-1). Comparison of the variants identified by our pipeline with the descriptions of the original publications showed that we could correctly recover the introduced mutations.

**Conclusions:**

Our pipeline offers researchers a fast and easy way to identify variants in the viral genome without additional genome sequencing. The pipeline is implemented as a workflow for the workflow management system Watchdog and is available at https://github.com/watchdog-wms/watchdog-wms-workflows/ (workflow VariantCallerPipeline).

## Introduction

Advances in molecular biology and genetics provide new technologies for studying virus infections and the role of individual viral genes during infection. This provides the basis for the development of treatments against virus infections or for their use as tools in genetic engineering, vaccine development, or gene therapy. ^1^ A common approach is the creation of mutant virus strains (see e.g. Ref. 2) containing single nucleotide polymorphisms (SNPs) or insertions or deletions (indels) of sequences that alter the functions of individual viral genes. For well-studied viruses like herpesviruses, such experiments have been conducted for decades. Consequently, many commonly used mutant strains have been generated decades ago, often before complete genome sequences of these viruses were available (e.g., in Refs. 3– 11 to list just a few examples). These have often been passed between laboratories and used for a multitude of experiments. However, the precise genome location of mutations or inserted sequences are often poorly documented and other undocumented mutations may have been introduced either with the original mutation or in the time since. Furthermore, even for recently created viral mutants, the description in the corresponding articles are often very limited and do not provide nucleotide positions (e.g. in Ref. 12). Moreover, even if the precise location of introduced mutations is known, it is often important to verify their presence, in particular if results from experiments do not meet expectations.

The standard approach to identify mutations in viral genomes is genome sequencing, ^13^ which requires separate experiments. However, due to advances in high-throughput sequencing technologies, analysis of virus gene functions is now commonly performed using sequencing-based functional genomics assays of virus-infected cells, such as RNA sequencing (RNA-seq), Assay for Transposase-Accessible Chromatin sequencing (ATAC-seq), or chromatin immunoprecipitation (ChIP) followed by sequencing (ChIP-seq) (e.g. in Refs. 14, 15). Since functional genomics experiments commonly also provide nucleotide coverage of viral genomes, though generally with very variable coverage, they afford the unique opportunity to identify viral variants directly in the experiment of interest without additional genome sequencing.

**Figure 1.**
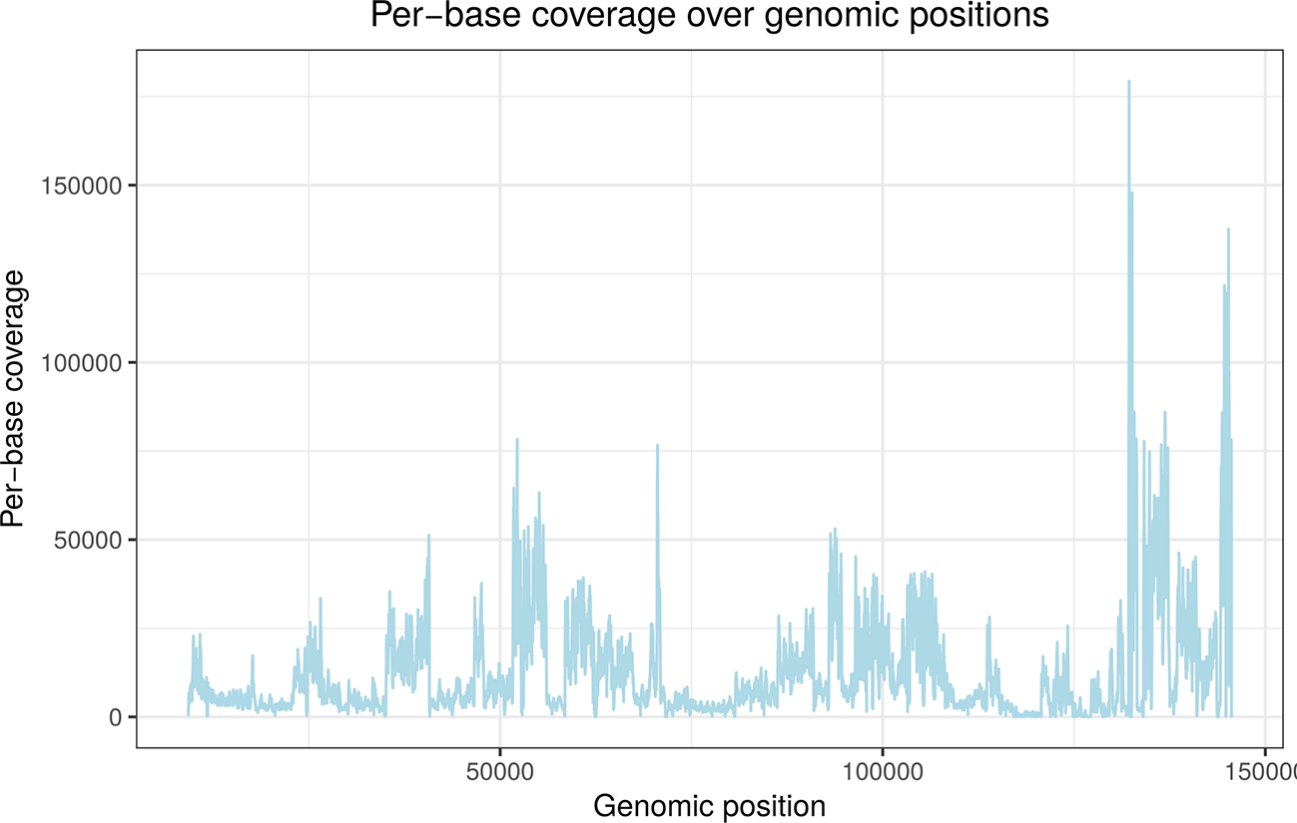
Per-base read coverage (y-axis) on the HSV-1 genome (x-axis) for a 4sU-seq sample for infection with an HSV-1 null mutant containing a deletion of the ICP22 protein (see Results for details). 4sU-seq is a variant of RNA-seq based on sequencing newly transcribed RNA obtained by 4-RNA labelling with thiouridine (4sU). ^30^ This shows that read coverage varies considerably across the genome depending on gene expression. The deletion is located between nucleotides 133,243 and 134,072 (see Table 1) but cannot be distinguished from other regions with low expression either visually or with standard deletion callers.

In this article, we present a pipeline to automatically identify viral variants in functional genomics data of virus infections, including SNPs, deletions and insertions and (optionally) inserted sequences. This pipeline uses existing SNP calling methods, in particular bcftools ^16^ and VarScan ^17^), which we found to perform well also for RNA-seq or other functional genomics data that exhibit large variations in read coverage across the viral genome (see e.g., Figure 1). In contrast, state-of-the-art structural variant callers we evaluated (DELLY, ^18^ GRIDSS2, ^19^ and BreakDancer ^20^) performed poorly in identifying insertions and deletions in viral null mutants from these data. This is not surprising, as RNA-seq data and other functional genomics data with non-uniform read distributions violate the underlying assumptions of existing structural variant callers. We thus implemented a new approach to identify deletions and insertions based on gaps in read coverage and clipped (i.e. partial) read alignments. We combined this with *de novo* assembly using rnaSPAdes ^21^ to identify inserted sequences. Analysis of previously published RNA-seq data for infection with knockout mutants of herpes simplex virus 1 (HSV-1) ^14^ and an HSV-1 strain expressing a green fluorescent protein (GFP) ^22^ showed that our pipeline allows fast and easy identification of viral variants and their precise genomic locations to characterize poorly documented mutant virus strains at the nucleotide level.

## Methods

### Implementation

The virus variant caller pipeline was implemented as a workflow for the workflow management system Watchdog and is available at https://github.com/watchdog-wms/watchdog-wms-workflows/ (workflow VariantCallerPipeline). The workflow takes as input read alignments against the viral genome in BAM format for one or more virus-infected samples. Read sequences in FASTQ format are only required if inserted sequences are to be identified, which is an optional step. We used BWA ^23^ for read alignment as it is very fast and requires little memory, but any read alignment program can be used that provides SAM/BAM output, includes read sequences in the output and produces clipped read alignments if only parts of a read can be aligned to the viral genome. Notably, since we are not interested in identifying splicing events, which are rare in viruses, there is no need to use a splicing-aware aligner for RNA-seq data.

The variant caller pipeline is divided into two main parts, which are described in the following: (1) SNP calling and (optionally) strain identification and (2) indel detection and (optionally) identification of inserted sequences.

***SNP calling***

**Figure 2.**
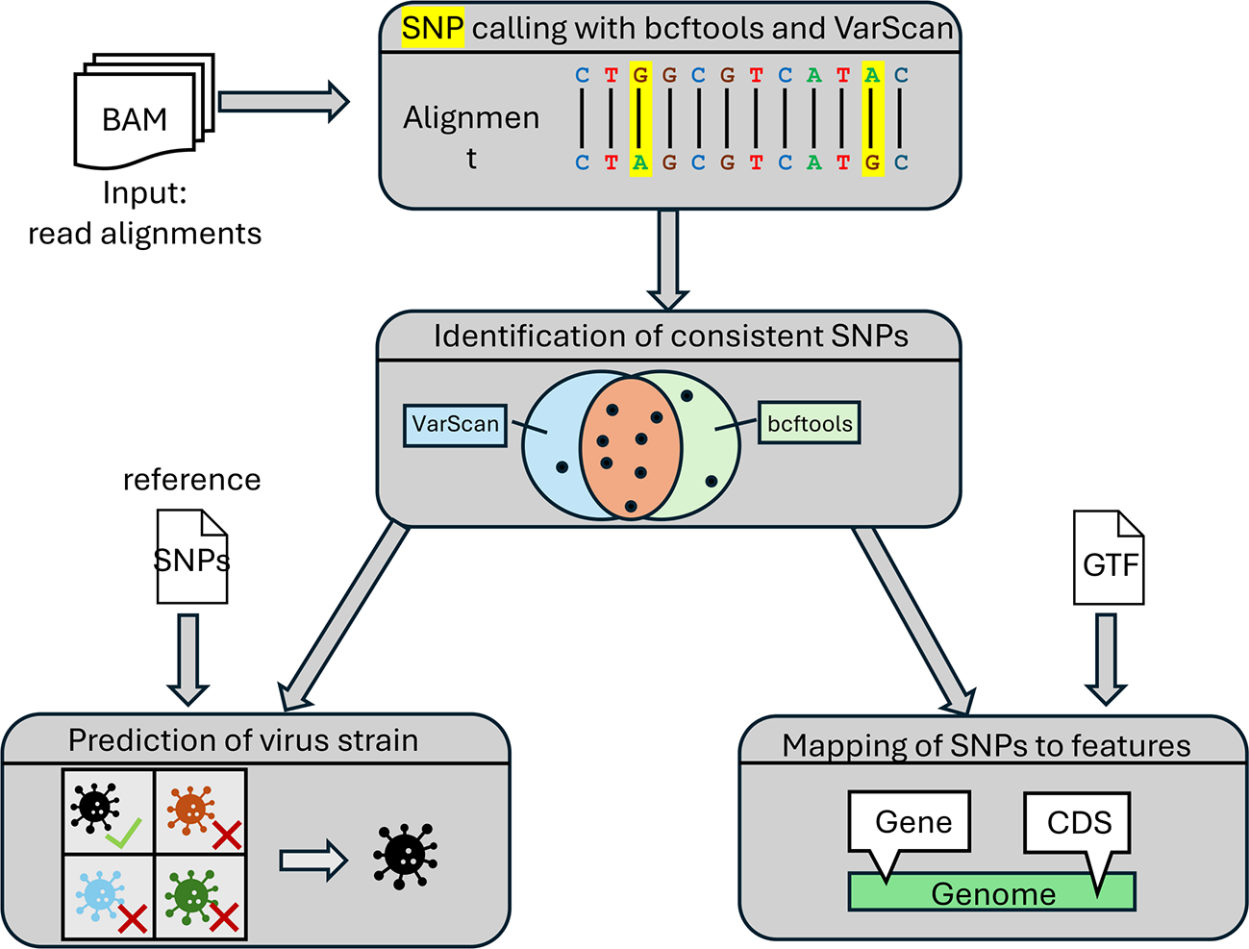
Overview of the steps the pipeline employs for SNP calling.

Figure 2 provides an overview of the steps performed for SNP calling. First, the variant callers bcftools ^16^ and VarScan ^17^ (after running ‘samtools mpileup’ ^24^) are applied independently to each input BAM file. Both tools provide the identified SNPs in the variant call format (VCF). ^25^ Next, so-called *consistent* SNPs are determined that are identified by both bcftools and VarScan. If more than one replicate is available, SNPs are considered consistent if they are detected by both tools in all replicates. Consistent SNPs are then mapped to viral features, e.g., genes, coding sequences, or introns, given a gene annotation in GTF format for the viral genome.

Furthermore, if a set of reference SNPs for different virus strains is provided by the user, the pipeline performs a prediction of the virus strain for each sample. This is useful both for verifying the virus strain used in the experiment and the parental strain from which a particular null mutant was generated. Such reference SNPs can be obtained by identifying consistent SNPs with our pipeline for functional genomics data of various virus strains. An example file with reference SNPs for HSV-1 strains 17, F and KOS 1.1 is included with example input files at https://doi.org/10.5281/zenodo.14266852 and the Watchdog module for strain identification (identifyStrain, available at https://github.com/watchdog-wms/watchdog-wms-modules).

For strain identification, the following distance D is calculated for each reference strain: $D=\left|S_{1}\cup S_{2}\right|-\left|S_{1}\cap S_{2}\right|$ , with $S_{1}$ the set of consistent SNPs identified for the virus used in the experiment and $S_{2}$ the set of reference SNPs for a reference strain. The strain with the smallest distance D is then predicted for the virus. This measure is largely independent of the reference genome sequence used for read alignment. For illustration, consider the following example. Assume a sample S that was derived from strain X but is aligned against the genome sequence of a different strain Y . Furthermore, reference SNPs for strains X , Y and Z were also obtained by aligning functional genomics data for these strains against the genome for strain Y . This will result in a (relatively) large number of consistent SNPs for sample S , no/few reference SNPs for strain Y and (relatively) large numbers of reference SNPs for strains X and Z . Since consistent SNPs for S and reference SNPs for X will be largely the same, the distance will be close to zero. The distance for Y and Z will be larger since consistent SNPs for S are not in the reference set for Y and will differ from reference SNPs for Z .

***Indel detection***

**Figure 3.**
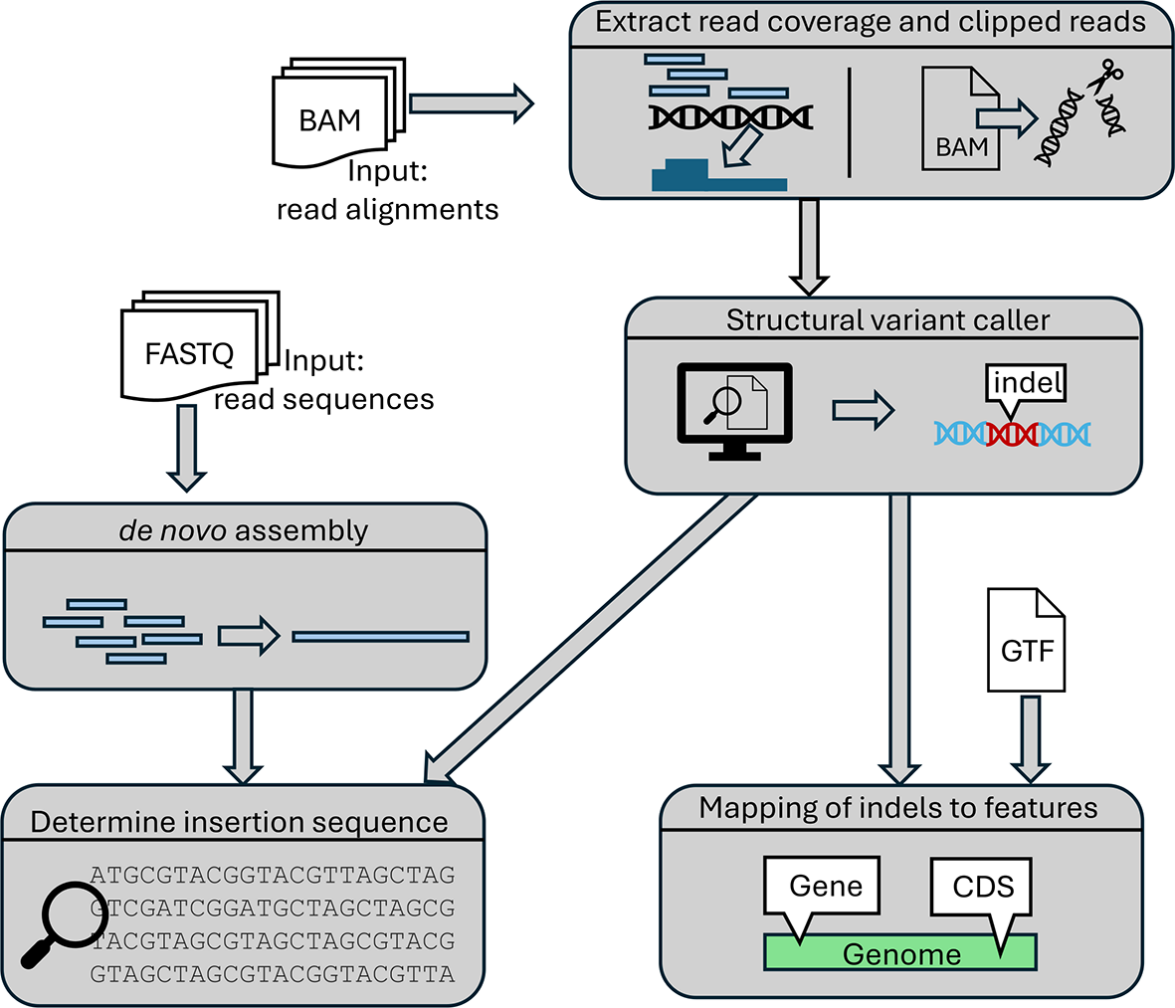
Overview of the steps the pipeline employs for indel detection.

Insertions and deletions in viral genomes are determined as outlined in Figure 3. First, per-base read coverage, i.e. the number of reads overlapping each genome position, and clipped reads (= reads with unaligned parts) are extracted from each input BAM file using samtools. ^24^ Since we are interested in indels in the genome, read coverage is determined in a non-strand-specific manner. Clipped reads are characterized by a CIGAR string matching the pattern “[0–9]+S.*” (left-clipped read) or “.*[0–9]+S” (right-clipped read), which indicate clipping at the start or end of the read, respectively. We included only soft-clipped reads, not hard-clipped reads (which contain an H instead of an S in the CIGAR string), as we further analyze the clipped parts of reads (see below) and these are only included in the BAM files for soft-clipped reads. Furthermore, BWA uses hard clipping only for supplementary alignments of reads for which parts of the read align elsewhere on the genome. The results are then used as input for indel calling as described below. Subsequently, identified indels are also mapped to genomic features. In addition, the pipeline can identify inserted sequences by combining the results from insertion detection with *de novo* read assembly obtained with rnaSPAdes ^21^ if raw read sequences in FASTQ format are provided.

*Candidate deletion detection*

The pipeline first detects potential deletions by identifying regions of the genome with very low read coverage compared to (i) the complete genome using a global threshold and (ii) the surrounding genomic regions using a local threshold. For this purpose, a global z-score is calculated for each position *i* in the genome. The global z-score is calculated as $\frac{\text{log}c_{i}-\mu }{\sigma }$ where *c*_*i*_ is the read coverage at position *i*, while *μ* and *σ* are the mean and standard deviation, respectively of the log read coverage for all positions in the genome. If *c*_*i*_ = 0, it is set to a user-defined pseudocount (default = 1). If the global z-score is below a stringent global threshold, the position is labelled as a potential deletion. If it passes only a less stringent global threshold, a local z-score is calculated as $\frac{\text{log}c_{i}-\mu_{l}}{\sigma_{l}}$ where *μ*_*l*_ and *σ*_*l*_ are the mean and standard deviation, respectively, of the log read coverage of the previous l nucleotides (nt) before the current position *i* (by default l=500 ). If the local z-score is below the local threshold, the position will also be labelled as a potential deletion.

The local z-score is used as read coverage can vary massively between positions in functional genomics data. This is exemplified for an RNA-seq sample in Figure 1. However, calculating local z-cores for every position is very costly as it requires calculating the mean and standard deviation over the preceding n nt for every genomic position. Thus, the stringent global z-score threshold is first employed to identify clear-cut cases of potential deletions. Local z-scores are only calculated for less clear-cut cases. Optionally, a user-defined length threshold can also be used to exclude very short deletions.

*Deletion verification*

**Figure 4.**
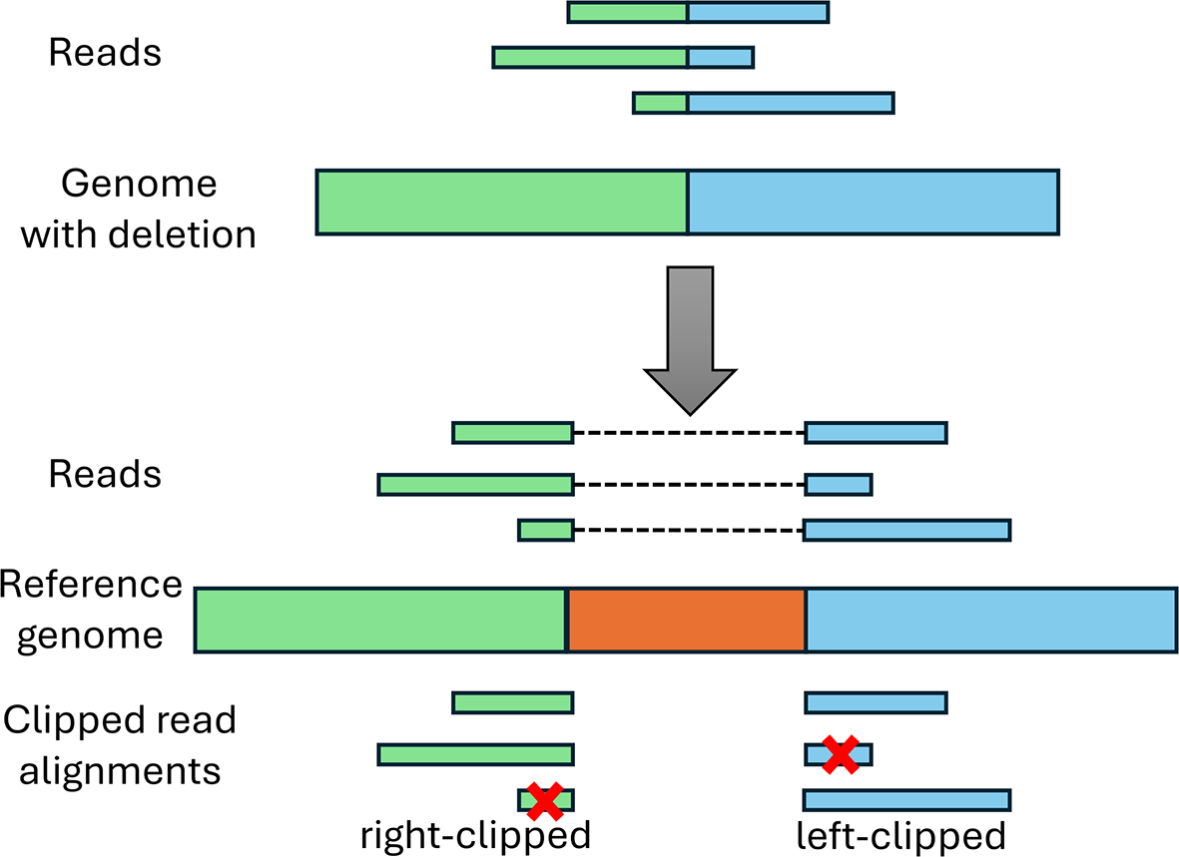
Illustration of clipping at deletion sites. The top shows the mutated viral genome that contains the green and blue sequences from the reference genome below, but the orange sequence was deleted. Reads from the mutant viral genome can thus only be aligned with gaps to the reference genome (top of the reference genome). If a non-splice-aware aligner is used or a splice-aware aligner that requires presence of splice signals, this results in clipped read alignments (at the bottom). If both parts of the read are sufficiently long to be aligned to the genome, this will result in multiple clipped alignments per read. If a part of the read is too short for alignment (marked by a red cross), this part will not be aligned at all.

Candidate deletions are subsequently verified using clipped read alignments. As depicted in Figure 4, reads crossing a deletion in the genome can only be aligned with gaps to the reference genome. If the alignment is performed using a non-splice-aware read aligner, such as BWA, this will result in clipped read alignments where only parts of the read are aligned to the genome. Notably, this often also occurs with splice-aware read aligners as the start and end nucleotides of the deletion generally do not match canonical splicing signals expected by many splice-aware read aligners.

Deletions should exhibit a peak of right-clipped reads ending at the deletion start and a peak of left-clipped reads beginning at the deletion end. Such peaks of clipped reads are again identified using both a global and local z-score, both of which are calculated separately for peaks of right-clipped and left-clipped reads. The global z-score at each position *i* is calculated as $\frac{c_{i}-\mu }{\sigma }$ , where *c*_*i*_ is the number of clipped reads of the particular type, i.e. either right- or left-clipped, ending or starting at position *i*, respectively, and *μ* and *σ* are the mean and standard deviation, respectively, of the number of clipped reads of the same type across the whole genome. For the local z-score, the mean and standard deviation are calculated only across all genomic positions within a window starting x nt upstream of the candidate peak and ending x nt downstream of the candidate peak, where *x* is a user-defined window size (by default x=20 ), excluding the peak position itself. If both the global and the local z-scores pass a global (default: 10) and local (default: 50) threshold, respectively, the position is considered a peak. In addition, a minimum number of reads is required for a peak (default: 10 reads).

To verify deletions, the pipeline identifies pairs of right-clipped and subsequent left-clipped peaks (i.e. the *clipping pattern* of deletions) and determines whether the positions of the two peaks overlap with a candidate deletion detected based on the per-base read coverage. Subsequently, the clipped sequences of the corresponding clipped read alignments (i.e. the unaligned part of the read in this alignment) are extracted from the BAM file and position-weight-matrices (PWMs) are computed from the sequence profiles of the clipped sequence parts. As can be seen in Figure 4, the PWMs of the clipped sequence parts on either side of the deletion should match the reference sequence on the opposite side of the deletion.

To test this, the best match of each PWM is determined in a window around the opposite deletion end. The score of a potential match is calculated as the sum of log-odds scores over all positions comparing the value of the PWM for the nucleotide at this position against the background probability of that nucleotide in the complete genome sequence. The best match for a PWM is the match with the highest score. If the best matches for both PWMs have a score >0 or at least one has a score >1, the deletion is accepted. If neither match is good enough, the deletion is flagged as a potential deletion that may contain an insertion. This special case was observed for one of the data sets analysed in the results section. In this case, the potential insertion sequence is determined as described in the next section and can be further analysed.

It should be noted that our approach for predicting deletions may also identify splicing events in RNA-seq data. However, splicing is rare in viruses and the few cases detected can easily be excluded based on the mapping to the genome annotation that is automatically performed for all detected variants. For instance, even a very thorough re-annotation of the HSV-1 genome, a relatively large viral genome of ~152 kb, based on short- and long-read RNA-seq identified only 15 splicing events. ^26^ Most of those had only low abundance compared to the corresponding unspliced transcripts and thus would not be recovered by our pipeline as read coverage does not drop significantly in the spliced part of the genome.

*Insertion detection*

**Figure 5.**
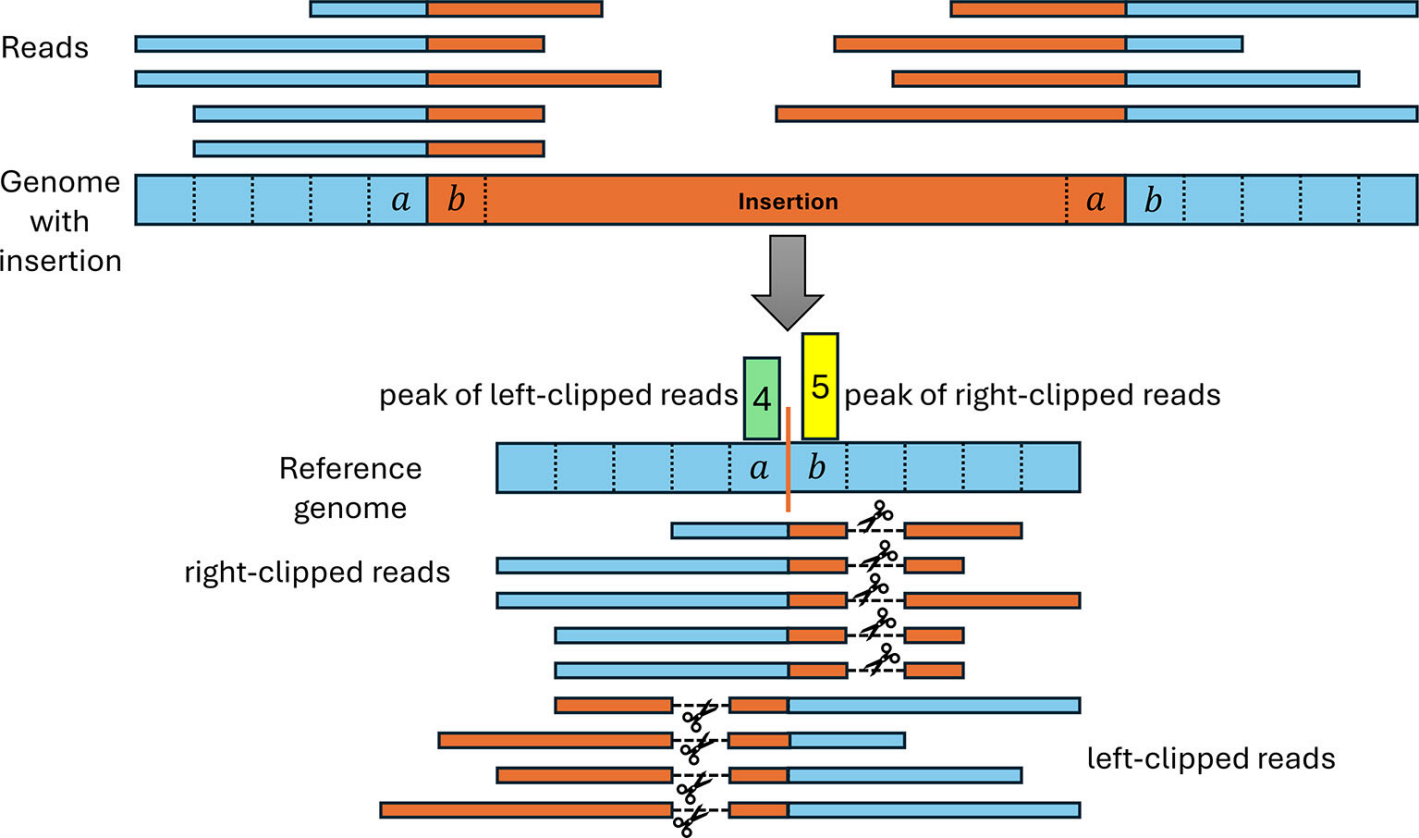
Illustration of read clipping at insertion sites. The top shows the mutated viral genome (blue) that contains an inserted sequence (orange) not present in the reference genome. Reads spanning the boundary of the insertion therefore contain parts of both the reference and insertion sequence. When aligned to the reference genome, the part of the reads containing the insertion sequence (orange) have to be clipped since they cannot be aligned to the reference genome. We observed that commonly the start and/or end of the insertion also matches the reference genome directly before and after the insertion site (in this example, 1 nt matches on each side). As a result, reads can be aligned beyond the insertion site, resulting in a distinctive insertion clipping pattern with a peak of left-clipped positions one or more positions left of a peak of right-clipped positions.

Our pipeline also uses clipped read alignments to determine insertions since reads containing part of an inserted sequence cannot be completely aligned to the genome (see Figure 5). Originally, we expected that the resulting clipping pattern should consist of a peak of right-clipped reads at a reference genome position (denoted as n ) preceding the insertion in the genome followed by a peak of left-clipped reads at the following position n+1 . However, the examples of null mutants created by insertions that we investigated showed a different pattern, consisting of a peak of left-clipped reads at position n and a peak of right-peaked reads at position n+1 (see Figure 5). This results from the first and last position of inserted sequences matching the genome on the other side of the insertion and is likely a consequence of the use of homologous recombination for inserting sequences. ^27^

To allow for such matches between the insertion start and/or end to the surrounding genomic regions, we introduced a parameter ϕ determining the maximum number of such matches that are allowed. Thus, any pair of positions for a left-clipped peak $p_{l}$ and a right-clipped peak $p_{r}$ is used to predict an insertion if $p_{r}-p_{l}+1\leq \phi$ . In the example in Figure 5, $p_{r}-p_{l}+1=2$ . For each identified insertion, we extract the non-aligned parts of clipped reads to calculate consensus sequences for the insertion start and end, respectively. These consensus sequences are commonly 30-40 nt long.

To identify the remaining central part of the inserted sequences, the pipeline optionally performs a *de novo* sequence assembly using rnaSPAdes, ^21^ a modification of the genome assembler SPAdes ^28^ for application to RNA-seq data. Assembly is performed for all reads, which also includes reads from non-viral sequences, in particular the inserted sequences. Following this, the consensus sequences for the insertion start and end are aligned to the resulting assembled contigs using BWA. If a match for both consensus sequences is found, the assembled sequence starting with the consensus of the insertion start and ending with the consensus of the insertion end is extracted. Insertion sequences containing only one of the consensus sequences are also extracted but are flagged for special attention. The origin of the inserted sequences can then be confirmed using BLAST. ^29^

We also investigated whether *de novo* assembly alone was sufficient for detection of both insertions and deletions by aligning the assembled contigs to the viral reference genome (see results). However, this either resulted in too few or too many indels depending on parameters, thus we did not pursue this approach for the pipeline.

### Operation

Watchdog and the VariantCallerPipeline can be run on Linux and MacOS systems. Running Watchdog requires Java 11 or higher. The deployment of required software during the VariantCallerPipeline run is performed with conda ( https://conda.io, using conda-forge and bioconda channels) using the deployment functionality of Watchdog. Watchdog also supports easy parallelization of workflow runs on computing clusters and monitoring of workflow execution, which can be used when running our pipeline. Example input and output files can be found at https://doi.org/10.5281/zenodo.14266852, including notes on software versions and expected runtime for each step. A detailed README on installing and running the pipeline can be found at https://github.com/watchdog-wms/watchdog-wms-workflows/ in the VariantCallerPipeline directory. A Docker image for running the pipeline (pre-configured to run the example) is available via Docker hub ( https://hub.docker.com/r/carolinefriedel/virus-variant-caller).

## Results

### Input data

We applied our pipeline to previously published 4sU-seq data for infection with null mutants for multiple HSV-1 proteins. ^14^ 4sU-seq is a variant of RNA-seq based on sequencing newly transcribed RNA obtained by RNA labelling with 4-thiouridine (4sU). ^30^ 4sU-seq was performed for null mutant viruses of the following HSV-1 proteins:

• ICP4, null mutant created from HSV-1 strain 17 by a SNP, which resulted in a temperature sensitive mutant (TsK) ^3, 5^
• ICP0 and ICP22, null mutants created by deletions from HSV-1 strains 17 and F, respectively ^4, 6, 7^
• ICP4, ICP27 and vhs, null mutants created by insertions from HSV-1 strains 17, KOS 1.1 and 17, respectively. ^8– 11^

The precise genomic location for these null mutants have not been described and most of these were created before the first HSV-1 genome sequence (for strain 17) was completed in 1988. ^31^ Two replicates were available for all null mutant viruses, except for the ICP4 knockout by insertion (ΔICP4), for which only one replicate was performed.

In addition, we analysed RNA-seq data for human brain organoids ^22^ infected with an HSV-1 strain 17 virus engineered to express GFP. ^12^ Here, RNA-seq data was available for brain organoids from two genetically distinct induced pluripotent stem cell lines, each infected for 3 and 6 days (2 replicates each, resulting in 8 samples).

All 4sU-seq and RNA-seq samples were aligned against the HSV-1 strain 17 genome (GenBank accession: JN555585) using BWA and then fed into the pipeline. The HSV-1 genome contains two repeat regions at each end of the genome that are repeated internally in the genome. Since read alignment cannot distinguish between the two repeats, one occurrence of each repeat (i.e. the ones at the genome ends) was replaced by N’s for read alignment.

The performance of the pipeline was evaluated by comparing the results with the descriptions of the original publications. The insertion sequences that were extracted by the pipeline from the sequence assembly were investigated with the NCBI BLAST webserver to identify their origin. Full results obtained with the pipeline on the 4sU-seq data and BLAST results are available at https://doi.org/10.5281/zenodo.17979981.

### SNPs in the TsK mutant

Our pipeline identified 28 consistent SNPs in the TsK mutant, three of which were in the ICP4 gene. One of these was consistent with the sequence change identified by Davison *et al.* ^5^ as responsible for the mutant phenotype: a replacement of a C:G base pair by a T:A base pair that changed the 475th codon of the ICP4 gene from an alanine codon to a valine codon. Our pipeline matched this missense mutation to a SNP at nucleotide 129,708. It furthermore showed that the TsK mutant differed from its parental strain 17 by an additional 27 SNPs, whose effects remain unclear. In particular, one of the other two SNPs identified in the ICP4 gene leads to a second amino acid change in ICP4 from serine to asparagine.

### Deletions identified for HSV-1 null mutants

To detect deletions, our pipeline was run with a stringent global z-score cut-off of -2.5, a less stringent global cut-off of 0.0 and a local z-score cut-off of -6.0. No minimum length was required for the deletions. This resulted in the identification of the deletions shown in Table 1. We identified a deletion each in the ICP0 null mutant (ΔICP0) and the ICP22 null mutant (ΔICP22), respectively, that matched the target gene and approximate length described in the corresponding articles. ^4, 6, 7^ Furthermore, the sequences found directly up- and downstream of the predicted deletions matched the target sequences of the restriction enzymes used in the corresponding experiments to create the deletions (XhoI & SalI for ΔICP0; PvuII & BstEII/Eco91I for ΔdICP22). Thus, we could recover the exact locations of the introduced deletions.

**Table 1.** Deletions detected by the pipeline for any of the HSV-1 null mutants in the 4sU-seq data and whether this represents the deletion described in the original papers describing the null mutant, a deletion in the parental strain or a known intron. The genomic context, i.e. the gene, its strand and the feature within the gene, in which the deletion is located is also described. For the known intron in ICP22, the position of the intron is also indicated in the last column. The genes US10-US12 overlap at the deletion position in the ΔICP27 virus. Coordinates of deletions are 1-based and inclusive.

Mutant	Type	Start position	End position	Size	Gene (strand, feature)
ΔICP0	described deletion	120913	123031	2119 nt	ICP0 (-, CDS & intron)
ΔICP22	deletion in parental strain F	132276	132280	5 nt	ICP22 (+, 5′UTR)
ΔICP22	described deletion	133243	134072	830 nt	ICP22 (+, CDS & 3′UTR)
ΔICP27	deletion in parental strain KOS 1.1	144838	144849	12 nt	US10;US11;US12 (-, CDS )
ΔICP27	part of known intron	132404	132497	94 nt	ICP22 (+, intron from 132,375-132,543)
Δvhs	part of known intron	132390	132513	124 nt	ICP22 (+, intron from 132,375-132,543)

A further deletion in the 5’ UTR of ICP22 identified in ΔICP22 infection corresponded to a genome deletion in the parental strain F from which the ΔICP22 virus was derived. Similarly, a deletion identified in the ΔICP27 virus is already present in its parental strain KOS 1.1. In addition, a deletion was identified for the ICP27 null mutant (ΔICP27) and the vhs null mutant (Δvhs) that fell into a known intron in the ICP22 gene. Although this intron is spliced in all samples, it was not detected in ΔICP0, ΔICP4 and TsK infection. For ΔICP4 and TsK infection this was likely due to the fact that read coverage on the whole viral genome was relatively low as ICP4 is necessary for optimal expression of other HSV-1 genes. ^32^ For ΔICP0 infection the opposite applied as both replicates had by far the highest read coverage on the viral genome of any of the samples. As a consequence, sufficient numbers of reads from unspliced ICP22 transcripts were detected for the intron not to be identified as a deletion.

### Insertions identified for HSV-1 null mutants

Insertions were also predicted using default values, i.e. at least 10 clipped reads were required for each peak and ϕ was set to 10. This means that a maximum overlap of 10 nt was allowed for the insertion ends and the surrounding genome regions. Local z-scores were calculated for the 40 nt around each peak position. Furthermore, the consensus sequences obtained from the clipped parts of the read had to be at least 10 nt long. The identified insertions are listed in Table 2.

**Table 2.** Insertions detected by the pipeline for any of the HSV-1 null mutants in the 4sU-seq data and whether this represents the insertion described in the original papers describing the null mutant or an insertion in the parental strain. Information in brackets indicates characteristics of the inserted sequences that could be confirmed from the consensus sequences or the assembly followed by BLAST. Overlap = overlap between the ends of the inserted sequence and the surrounding genome sequences. The genes US5-US7 overlap at the insertion position in the parental strain KOS 1.1. The genomic context, i.e. gene, its strand and the feature within the gene, in which the insertion is located is also described. Coordinates of insertions are 1-based.

Mutant	Type	Position	Overlap	Size	Gene (strand, feature)
ΔICP4	described insertion (stop codons, HpaI recognition site)	130376	4	16 nt	ICP4 (-, CDS)
ΔICP27	described insertion (E. coli lacZ gene)	113648	3	≥3145 nt	ICP27 (+, CDS )
ΔICP27	insertion in deletion in parental strain KOS 1.1	140458	3	unclear	US5;US6;US7 (+, CDS)
Δvhs	described insertion (cloning vector with lacZ gene)	91923	2	≥4132 nt	vhs (-, CDS)

All but one of the identified insertions matched the description in the corresponding publications on how the null mutants were created. ^8– 11^ In particular, we could confirm the insertion of lacZ genes in both the ΔICP27 and Δvhs virus by BLASTing the predicted insertion sequences obtained from the assembly. For both viruses, two insertion sequences each were assembled that were largely identical (>73%) but of different length. For the ΔICP4 mutant, the insertion of a small 16 nt sequence could be directly confirmed from the consensus sequences of the insertion start and end as these overlapped. This insertion sequence contained the 3 stop codons, one for each frame, and a recognition site of the HpaI restriction enzyme described in the original publication. The additional insertion identified in the ΔICP27 virus was less clear to interpret as it was contained within a 21 nt region already deleted in the parental strain KOS 1.1, which was one half of a tandem repeat in the strain 17 genome. Although 5 sequences were assembled whose start matched the consensus sequence obtained from the clipped reads for the insertion start, none of these matched the consensus sequence for the insertion end.

Interestingly, we found that the position for the insertion in the vhs coding sequence (251st codon) described in the corresponding publication for the Δvhs mutant ^10^ may have been calculated based on a wrong strand assignment. The vhs gene is located on the negative strand, with the coding sequence ranging from positions 91,167 (stop codon) to 92,636 (start codon). Accordingly, the insertion position identified by our pipeline (91,923) is in the 238th codon. However, if the codon position is erroneously calculated from the positive strand, the insertion would be after the first position of the 252nd codon (excluding the stop codon), which is closer to the original publication. Since the insertion site was described to be in the unique recognition site of the NruI restriction enzyme in the vhs gene ^10^ and the centre of this NruI recognition site is at the insertion position identified by our pipeline, this is indeed the correct position.

### Insertions in the GFP-expressing HSV-1 virus

According to the original publication describing this virus, ^12^ an enhanced GFP (EGFP) gene with a mouse cytomegalovirus promoter was inserted between the open reading frames (ORFs) UL55 and UL56. In addition, a LoxP site (= a 34 nt DNA sequence recognized by the Cre recombinase enzyme) was inserted downstream of the UL23 ORF. Two insertion sites at positions 46,665 and 116,147 were identified in 8 and 7 of the samples, respectively, located downstream of the UL23 coding sequence and between UL55 and UL56, respectively. The insertion sequence for the first insertion indeed contained a LoxP site and BLAST analysis of the insertion sequence for the second insertion site showed that it matched several cloning vectors containing the GFP gene. Thus, we correctly identified the precise genome positions of both insertions.

It should be noted that the insertion at position 116,147 was actually identified as a deletion between positions 116,147 and 116,154 into which an insertion was placed. This special case is predicted by the pipeline if the PWMs obtained from the clipped reads cannot be matched to the opposite end of the deletion and an insertion sequence can be identified from the assembly. Unfortunately, the description in the original publication on how the sequence was inserted is not sufficiently detailed to explain how this small deletion was generated during the insertion process, but it is most likely a consequence of the experimental approach used.

Additional insertions were identified at positions 62,143, 106,984, and 119,496 in 4-8 of the samples. However, no insertion sequences could be extracted from the assembly for insertions at positions 62,143 and 106,984 based on the consensus sequences from the clipped reads, while the insertion sequence for 119,496 matched the genome downstream of the predicted insertion site. Based on these results and inspection of the genome at these positions, we concluded that these represented artefacts from repetitive sequences. This highlights how the combination of consensus sequences from the clipped parts of reads and the assembly can be used to filter out incorrectly identified insertions.

### Comparison to *de novo* assembly

For comparison, we also investigated whether deletions and insertions could be identified directly from the contigs assembled by rnaSPAdes instead of performing the analysis of read coverage and clipped read alignments performed by our pipeline. For this purpose, contigs assembled for the 4sU-seq data of HSV-1 mutant infections were aligned against the reference genome using minimap2. ^33^ However, this showed that assembled contigs often contained small indels (~1-50bp) compared to the reference genome, which would result in a large number of predicted indels if we included all of them. Thus, we evaluated different minimum length thresholds on identified indels. Furthermore, we observed that for some insertions the inserted sequence was only partially assembled and thus located at the start or end of assembled contigs. This resulted in a clipped alignment of these contigs to the genome. We thus also evaluated the option to include such clipped alignments to identify the position of the insertion and at least the start or end of the inserted sequence.

**Figure 6.**
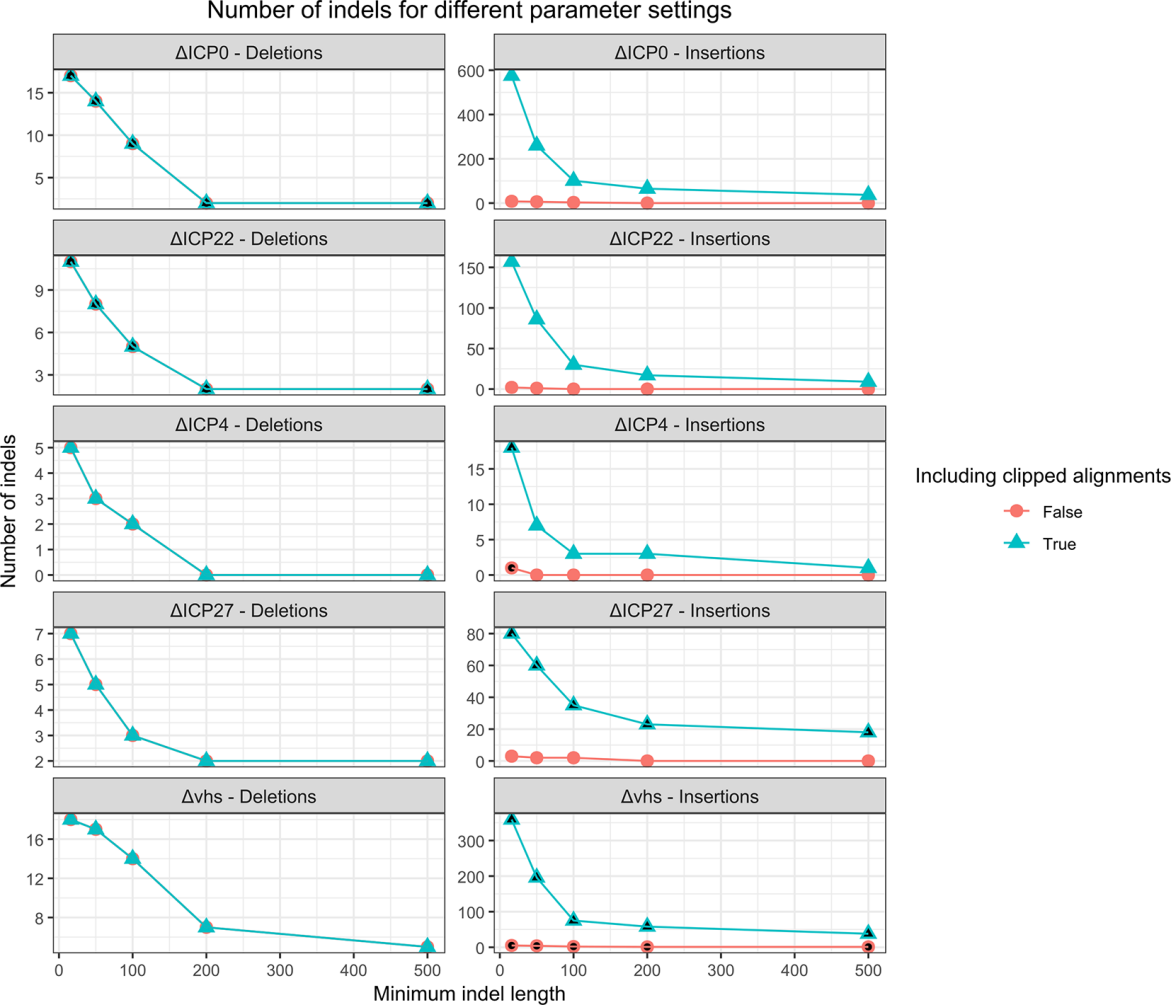
Analysis of the number of predicted deletions or insertions identified from the contigs assembled from the raw sequencing reads for different minimum indel lengths. For insertions, we also evaluated the effect of predicting insertions if only one end of the contig can be aligned to the genome in a clipped alignment. Parameters for which the correct deletion (for the ΔICP0 and ΔICP22 viruses) or the correct insertion (for the ΔICP4, ΔICP27 and Δvhs viruses) is recovered are filled in black.

Figure 6 shows an evaluation of different thresholds on the indel length with and without inclusion of clipped contig alignments for insertion detection. This showed that a relatively small minimum indel length of 16 nt had to be used to identify all indels and clipped contig alignments had to be included. Higher minimum indel lengths excluded the 16 nt insertion in the ΔICP4 mutant, while the lacZ gene insertion in the ΔICP27 mutant would be missed without allowing clipped contig alignments. However, this parameter combination resulted in large numbers of predicted insertions for ΔICP0, ΔICP22, ΔICP27, and Δvhs mutants, making it difficult to distinguish the correct indels in these mutants.

## Discussion

In this article, we present a pipeline for identification of SNPs and indels in viral variants from functional genomics experiments, such as RNA-seq, ATAC-seq or others. Development of the pipeline was motivated by the observation that commonly used null mutant viruses are often not described in sufficient detail to determine the precise genomic location of mutations. Notably, this does not only apply to null mutants created decades ago before the availability of viral genome sequences but also to more recently created virus variants as the GFP-expressing HSV-1 virus from. ^12^ In the latter case, only the approximate location relative to viral genes was described. Thus, application of our pipeline provides the first annotation of the precise genome location for key mutations in several widely used HSV-1 mutant viruses.

Our pipeline has the advantage that it does not require additional genome sequencing experiments and can be run directly on the experiment from which biological conclusions are drawn. Furthermore, the computational overhead is relatively low, in particular if sequence assembly for identification of longer insertion sequences is omitted. This would be sufficient if one is not interested in the insertion sequence or the insertion is short enough that the sequence can be identified directly from the consensus sequences as in the case of the ΔICP4 mutant.

Without assembly, indel detection runs in a few minutes for one sample instead of > 1h with assembly, reducing the runtime enormously. For SNP detection, computational overhead is determined by the runtimes of bcftools and VarScan (including ‘samtools mpileup’), which took about 20 and 15 minutes per sample, respectively, even for the ΔICP0 infection samples with the highest coverage of the HSV-1 genome.

Despite the additional overhead, identification of inserted sequences from read assemblies has the advantage that it allows confirming the insertion of particular marker genes like GFP or lacZ and distinguishing the marker insertions from other insertions that may have been correctly or incorrectly predicted. Notably, *de novo* assembly alone is not sufficient to identify indels with high precision without further post-processing and tuning parameters to a particular sample. In contrast, one parameter combination for our pipeline recovered all variants introduced into the HSV-1 null mutants without predicting too many additional indels. Notably, the additional indels identified by our pipeline for the HSV-1 null mutants were not actually incorrect as they represented indels in the parental strains of the null mutants or introns.

A disadvantage of our pipeline is that it depends on sufficient read coverage of the corresponding genome regions. While this also applies to standard genome sequencing, functional genomics data can have low read coverage either in parts or on the complete genome if they depict gene expression (such as RNA-seq, PRO-seq or similar methods to capture transcriptional processes) or if the viral genome shows generally low coverage. Although most parts of viral genomes are generally transcribed to some degree, lowly expressed genes or non-transcribed regions can have insufficient coverage. Read coverage can be low on the whole genome when virus genome replication and transcription are impaired, such as during ΔICP4 and TsK infections, or in the early stages of infection. Nevertheless, this issue can be addressed by combining different types of functional genomics data, replicates or different time points of infection.

Although we only tested the pipeline for (variants of ) RNA-seq data, these represented both the major challenges for our pipeline, i.e. variable and low coverage samples, and the most commonly applied assay for functional studies of viral null mutants. We are thus confident that our pipeline will be highly useful for researchers using functional genomics to study viruses and the functional role of individual virus genes.

## Data Availability

The data sets supporting the results of this article are available in the Gene Expression Omnibus (GEO) under the following identifiers:
• 4sU-seq data of HSV-1 null mutant infections: GSE151912, https://www.ncbi.nlm.nih.gov/geo/query/acc.cgi?acc=GSE151912 (previously published RNA-seq data from the study by Wang *et al.* ^14^). • RNA-seq data of infection with the GFP-expressing HSV-1 virus: GSE163952, https://www.ncbi.nlm.nih.gov/geo/query/acc.cgi?acc=GSE163952 (previously published RNA-seq data from the study by Rybak-Wolf *et al.* ^22^). A pre-print version of this article has been deposited at bioRxiv at: https://doi.org/10.1101/2025.01.31.635891. ^34^

## Software availability

Software available from: *https://github.com/watchdog-wms/watchdog-wms-workflows* , *https://github.com/watchdog-wms/watchdog-wms-modules*

A Docker image for running the pipeline (pre-configured to run the example at https://doi.org/10.5281/zenodo.14266852) is available from Docker hub: *https://hub.docker.com/r/carolinefriedel/virus-variant-caller*

Source code available from: *https://github.com/watchdog-wms/watchdog-wms-workflows* , *https://github.com/watchdog-wms/watchdog-wms-modules*

Archived source code at time of publication: *https://doi.org/10.5281/zenodo.16639950*

License: *GNU General Public License v3.0*
