# Supplementary material for: Identification of Viral Variants from Functional Genomics Data
Source: F1000Res. 2025 Dec 31;14:794. Originally published 2025 Aug 18. [Version 2] doi: 10.12688/f1000research.168786.2 (PMC13090762; doi:10.12688/f1000research.168786.2)
Supplement: Peer review history

# Reviewer report: Reviewer response for version 2

**DOI:** 10.5256/f1000research.194060.r469692

**Published:** 2026-04-17

**Authors:** Daniel P Depledge

**Affiliations:** 1 Institute of Virology, Hannover Medical School, Hannover, Germany

This manuscript tackles an interesting and important concept, namely the use of RNA-Seq datasets to reconstruct expressed regions from viral genomes with the aim of identifying undocumented mutations. The rationale for the work presented is robust, as is the implementation itself. The manuscript is clearly written and the figures well presented. Specific critiques are listed below.

 1. The final paragraph of the ‘deletion variants’ section in the methods states that ‘splicing is rare in viruses’ which is not accurate. While splicing in HSV-1 is indeed rare, other herpesviruses demonstrate higher splicing rates (HCMV, MDV, EBV). For adenoviruses (another nuclear dsDNA virus) the majority of viral transcripts are extensively spliced and it is not clear that the approaches proposed here would be compatible with adenovirus RNASeq datasets. Ideally, the authors whould demonstrate that their approach also works for additional viral species that are heavily spliced.  

 2. How does the methodology detailed here handle differences between viral RNAs and the underlying viral genome that are induced by changes to the RNA itself i.e. ADAR editing or installation of other modifications (e.g. pseudouridine) which would be represented as mismatches (A->G and C->T, respectively). The presence of modifications and/or editing events could lead to the interpretation of mixed populations or mutation in the genome that do not actually exist. The authors should acknowledge this issue and make the point that in some cases, differences observed may not reflect true variation in the underlying genome.

 3. While the potential utilities of this method are clear, there is a risk of this approach encouraging bad practices such as not verifying the underlying viral genome sequence before conducting experiments and sequencing. While this approach is clearly valuable for analyzing datasets with incomplete histories, it would be beneficial to highlight that this post-hoc approach should only be used as a replacement for genome sequencing in situations where sequencing the original viral genome is not possible.

 In addition to the above, I also have two more technical comments.

 1. Which parameters are being used in samtools mpileup? The --max-depth and -B/-C flags have significant impacts on the output and it explicitly stating and justifying the parameters chosen would be a benefit to the reader.

 2. In the Results->Input date section, the authors state that they replaced the sequence of one of the two terminal repeats with Ns. Is there a specific advantage to doing this over simply removing one of the terminal repeat sequences?

Are the conclusions about the tool and its performance adequately supported by the findings presented in the article?

Partly

Is the rationale for developing the new software tool clearly explained?

Yes

Is the description of the software tool technically sound?

Yes

Are sufficient details of the code, methods and analysis (if applicable) provided to allow replication of the software development and its use by others?

Yes

Is sufficient information provided to allow interpretation of the expected output datasets and any results generated using the tool?

Yes

Reviewer Expertise:

Molecular virology, RNA biology, Computational biology, Bioinformatics, RNA and DNA sequencing

I confirm that I have read this submission and believe that I have an appropriate level of expertise to confirm that it is of an acceptable scientific standard, however I have significant reservations, as outlined above.

# Reviewer report: Reviewer response for version 1

**DOI:** 10.5256/f1000research.185998.r420733

**Published:** 2025-11-03

**Authors:** Anuj Kumar

**Affiliations:** 1 Dalhousie University, Halifax, Canada

In my opinion, the authors have done an excellent job in developing a robust pipeline for screening viral variants from functional genomics data. The pipeline facilitates SNP calling, indel detection, candidate deletion identification, and additional variant analyses. Its user-friendly design makes it a valuable tool for the scientific community to efficiently identify and interpret viral genome variants.

Are the conclusions about the tool and its performance adequately supported by the findings presented in the article?

Yes

Is the rationale for developing the new software tool clearly explained?

Yes

Is the description of the software tool technically sound?

Yes

Are sufficient details of the code, methods and analysis (if applicable) provided to allow replication of the software development and its use by others?

Yes

Is sufficient information provided to allow interpretation of the expected output datasets and any results generated using the tool?

Yes

Reviewer Expertise:

Bioinformatics, Emerging infectious diseases, Functional Genomics

I confirm that I have read this submission and believe that I have an appropriate level of expertise to confirm that it is of an acceptable scientific standard.

# Reviewer report: Reviewer response for version 1

**DOI:** 10.5256/f1000research.185998.r408633

**Published:** 2025-09-19

**Authors:** Dóra Tombácz

**Affiliations:** 1 University of Szeged, Szeged, Csongrád, Hungary

**Full report**

 The manuscript presents a workflow (“VariantCallerPipeline”) for identifying viral SNPs and indels—including reconstruction of inserted sequences—directly from functional-genomics data (e.g., RNA-seq/4sU-seq) of infected cells, obviating separate viral genome sequencing. SNPs are called with bcftools and VarScan; indels are detected using a combination of read-coverage troughs and peaks of left/right clipped reads, with PWM-based breakpoint validation; optional rnaSPAdes assembly retrieves inserted sequences. On well-known HSV-1 mutants (ΔICP0, ΔICP22, ΔICP27, Δvhs, ΔICP4; TsK) and a GFP-expressing strain, the pipeline recovers the intended edits and clarifies additional parental-strain variants or introns. The problem is real and common—legacy mutants with sparse nucleotide-level documentation—and the solution is practical and timely.

**Strengths**

- Clear rationale; addresses a frequent, under-served need in virology labs.
- Sensible method design for uneven coverage typical of RNA-derived data.
- Convincing case studies on canonical HSV-1 mutants; insertion sequences verified (e.g., lacZ/EGFP).

**Limitations **

- Reliance on sufficient local read coverage; potential ambiguity with splice junctions in RNA-derived data if not controlled.
- Evaluation centered on HSV-1 RNA/4sU-seq (coverage profiles may differ in other assays/viruses).

**Points that must be addressed  **

1. Please pin exact software versions (bcftools/htslib, VarScan, samtools, BWA, SPAdes/rnaSPAdes), provide checksums for the reference genome and annotation used, and supply either a container (Docker/Singularity) **or** a one-command runnable example using your Zenodo inputs, with expected outputs and brief runtime/memory notes.
*Rationale:* ensures others can rerun the workflow without environment drift.
2. In Methods, specify how zeros are handled in log-coverage (pseudocount), define the global/local z-score calculations and clip-peak criteria, and add one sentence on preventing splice junctions being misinterpreted as deletions (e.g., default masking of annotated introns or an option for splice-aware alignment).
*Rationale:* removes ambiguity for RNA-derived datasets and documents default behavior.
3. Ensure Tables 1–2 list complete coordinates, event sizes, strand, and genomic context (CDS/UTR/intron). Deposit per-sample final VCF/BED (SNPs and indels) and FASTA for insert consensus sequences (with brief BLAST summaries).
*Rationale:* makes the results reusable and easy to interpret by others.

**Minor edits**

- Figure 1 caption: “a 4sU-seq sample” (not “an”).
- Correct typo: reference 27 author “Waldman”.
- Consistent notation: define n,x,ϕn, x, \phin,x,ϕ and coordinate conventions (1-based; inclusive bounds). Replace “local z-cores” with z-scores.

**Recommendation**

 The tool is valuable and technically sound; the three requested clarifications and packaging steps will make it easily reusable by the community

Are the conclusions about the tool and its performance adequately supported by the findings presented in the article?

Yes

Is the rationale for developing the new software tool clearly explained?

Yes

Is the description of the software tool technically sound?

Yes

Are sufficient details of the code, methods and analysis (if applicable) provided to allow replication of the software development and its use by others?

Partly

Is sufficient information provided to allow interpretation of the expected output datasets and any results generated using the tool?

Yes

Reviewer Expertise:

viral transcriptomics, genomics, metagenomics

I confirm that I have read this submission and believe that I have an appropriate level of expertise to confirm that it is of an acceptable scientific standard, however I have significant reservations, as outlined above.

# Response

**Published:** 2025-12-19

**Authors:** Caroline C. Friedel

**Affiliations:** Ludwig Maximilian University of Munich, Germany

Thank you for this generally positive assessment. We addressed all raised issues as outlined in the following:

 1.    A Docker image for running the pipeline (preconfigured to run the examples) is now available via Docker hub ( https://hub.docker.com/r/carolinefriedel/virus-variant-caller). Files for creating the Docker image are now also available together with the example files at Zenodo ( https://doi.org/10.5281/zenodo.14266852).

 Output files resulting from running the example are now also available at https://doi.org/10.5281/zenodo.14266852. This includes exact software versions and notes on runtime for all steps. Memory requirements are also indicated in the description of the Zenodo entry. All steps of the pipeline can be run on a laptop with 16 GB RAM, except for the assembly of inserted sequences with rnaSPAdes, which can be omitted. rnaSPAdes can be run with 32 GB RAM.

 Md5 checksums for the reference genome and annotation are now also included with the example. 

 2.    We uploaded a revised manuscript in which we specify how zeros are handled in log coverage, i.e. using a user-defined pseudocount (default 1),  and define the global/local z-score calculations and clip-peak criteria. We also added more information on how splice junctions are prevented as being misinterpreted as deletions, i.e. by automatically comparing them to the genome annotation. 

 3.    Tables 1-2 now also list the strand of the gene and the corresponding feature of this gene (i.e. CDS, UTR or intron) the corresponding deletion or insertion is contained in. We also clarified in the text that strand is not considered for indel detection since we determine indels present in the DNA, i.e. on both strands. The size of the deletion or insertion is also provided in the table, however for the ΔICP27 and Δvhs viruses this is only approximate as multiple, largely identical, insertion sequences were assembled. 

 The full pipeline output for the 4sU-seq data is now also available at Zenodo ( https://doi.org/10.5281/zenodo.17979981) together with the BLAST results for the assembled sequences. Output format are now also described in the description of the Zenodo entry and the README file available for the workflow at https://github.com/watchdog-wms/watchdog-wms-workflows. 

 4.    Minor edits: We corrected the two typos and defined the variables requested. Local z-scores was not changed to z-scores, as we calculate both global and local z-scores and we want to avoid confusion between the two. We added explanations to the tables that coordinates are 1-based and inclusive.
